# Exposure to thermal extremes favors higher solar reflectivity in intertidal gastropods

**DOI:** 10.1016/j.isci.2022.105674

**Published:** 2022-11-25

**Authors:** Amanda M. Franklin, Katrina J. Rankin, Andrew Hugall, Devi Stuart-Fox

**Affiliations:** 1 School of BioSciences, The University of Melbourne, Parkville, VIC 3010, Australia; 2 Sciences Department, Museums Victoria, Carlton, VIC 3053, Australia

**Keywords:** Ecology, Aquatic science, Zoology, Evolutionary biology

## Abstract

During low tides, intertidal animals can be exposed to extreme temperatures that can exceed the animals’ thermal limits. Reflectance of solar radiation could be critical to prevent overheating for animals exposed to the sun; however, most studies ignore near-infrared (NIR) wavelengths that comprise approximately half of solar energy. Here, we conduct a phylogenetically controlled analysis to test whether the reflectivity of intertidal gastropod species is associated with solar exposure. Gastropods from exposed microhabitats had greater shell total reflectivity than those from sheltered microhabitats. Dry shells of gastropods from exposed microhabitats had higher NIR reflectivity even after controlling for UV-visible reflectivity, supporting selection for thermal benefits independent of visual benefits. Using thermal imaging, we also demonstrated that gastropods with high shell reflectivity had lower heating rate in natural conditions than those with low shell reflectivity. Together, these studies show that reflectivity can play a crucial role in thermoregulation in extreme environments.

## Highlights

• Shell reflectivity is higher for intertidal gastropods from exposed microhabitats
• Higher shell reflectivity reduced gastropod heating rate in natural conditions
• Results support selection for thermal benefits of high reflectivity, independent of visual benefits

## Graphical abstract

**Figure:**
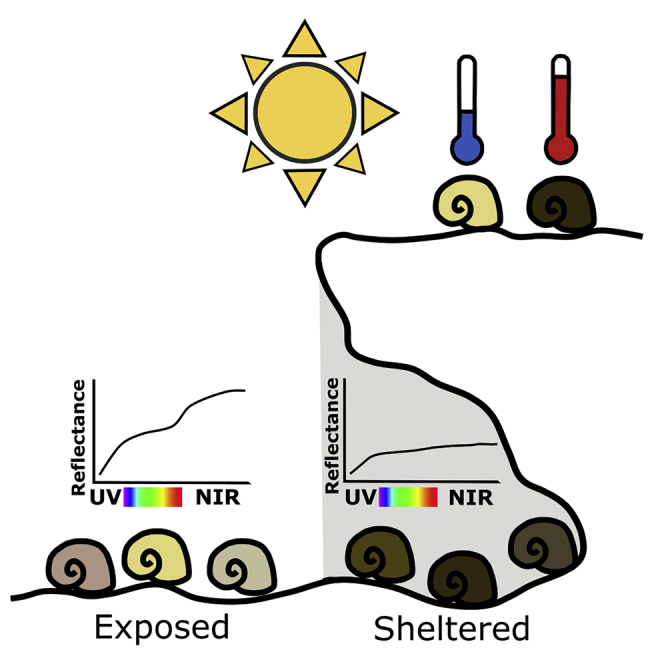

## Introduction

Remaining within critical thermal limits is fundamental to the survival of all organisms. As extreme weather events become more frequent because of climate change, adaptations to maintain body temperature within thermal limits are likely to play a pivotal role in survival.^1^^,^^2^^,^^3^ Behavioral adaptations, such as moving to shaded areas, can substantially reduce the impact of temperature extremes.^1^^,^^4^ However, sessile or slow-moving animals often cannot move out of direct sunlight and instead must rely on other physiological, morphological and behavioral adaptations.^5^^,^^6^ For example, tropical intertidal oysters (*Isognomon nucleus*) substantially lower their metabolic rate to reduce energetic demands when temperatures are high.^7^ Reflectance of exposed body parts is another adaptation that can lower heat gain by reducing the amount of solar energy absorbed.^8^^,^^9^^,^^10^^,^^11^ Although the sun emits ultraviolet-visible (UV-visible; 300–700 nm) and near infrared (NIR; 700–2500 nm) energy in approximately equal amounts, most studies ignore NIR reflectance despite the important role it can play in thermoregulation (for examples see ^8^^,^^12^^,^^13^)

NIR reflectance should be primarily selected for thermoregulatory requirements unlike UV-visible reflectance that will experience multiple selection pressures.^9^^,^^12^ Both UV-visible and near-infrared wavelengths influence heat gain, proportional to the absorption or reflectance of the integument.^8^^,^^14^ However, unlike UV-visible reflectance, variation in NIR reflectance is likely primarily driven by thermoregulatory requirements because most animals cannot see NIR wavelengths.^15^ This is because photoreceptors shifted to longer wavelengths are more readily activated by thermal noise, likely placing an upper limit on long wavelength sensitivity.^15^^,^^16^ On the other hand, variation in UV-visible reflectance will be driven by visual functions, such as communication and camouflage, as well as thermoregulatory functions. These different selective pressures can lead to a stronger association between climate and NIR reflectance than climate and UV-visible reflectance.^12^ To date, these trends have only been investigated in a few taxa (birds: Medina et al. 2018^17^; butterflies: Kang et al. 2021^13^, Munro et al. 2019^12^) and have not been investigated on a smaller scale, such as between microhabitats.

One community that can experience extreme temperatures is intertidal organisms. During summer low tides, intertidal organisms can be exposed to air temperatures significantly higher than the water temperature. For example, a 35°C summer day in south-eastern Australia will be roughly 15°C hotter than seawater temperature.^18^ On extremely hot days this sun exposure can put intertidal animals close to or above their lethal thermal limits,^19^^,^^20^ which can lead to mass mortalities.^21^ Although some intertidal animals can move to rock pools or crevices out of the sun, many are sessile or slow moving and remain exposed to the sun during low tides.^5^^,^^6^ Immediately after emersion, these animals will be wet and benefit from evaporative cooling.^22^ Even though wet surfaces are generally darker than dry surfaces,^23^^,^^24^ the heat lost from evaporative cooling will likely outweigh heat gain from absorption.^25^ However, on warm to hot days surfaces can dry rapidly and at this point radiative heat gain can be a significant problem for exposed animals.^25^^,^^26^ For these animals high reflectance may play a key role in minimizing heat gain.^27^ In fact, intertidal gastropods found in hotter climates or higher in the intertidal zone (i.e., exposed for longer) often have lighter shells.^28^^,^^29^^,^^30^ Whether this relationship extends to NIR reflectance is unknown, even though NIR reflectance could play a crucial role in reducing the risk of overheating.

Here, we investigate whether reflectivity correlates with microhabitat for intertidal gastropods. Gastropods in exposed positions in the intertidal zone will be most susceptible to overheating because they will be exposed to more solar radiation and their shells will dry more quickly than gastropods in sheltered locations (e.g., in crevices or under rock overhangs). Therefore, we would expect that gastropod species predominantly in exposed locations (i.e., higher in the intertidal zone or in exposed positions), will have higher reflectivity and a greater increase in reflectance as they dry, compared to those in sheltered locations (i.e., lower in the intertidal zone or in sheltered positions). However, other functions of color could lead to the same predictions. Gastropods in sheltered locations may be darker than those in exposed habitats to camouflage more effectively in the shaded, darker environment. To distinguish between these two drivers of reflectance, we can investigate whether the relationships are present within NIR wavelengths because NIR wavelengths are likely not visible to predators of intertidal gastropods (e.g., birds, crustaceans, and fish ^15^^,^^16^). If dry shells of exposed gastropods have higher relative NIR reflectance (accounting for UV-visible reflectance), this indicates that NIR reflectance has been selected for minimizing heat gain.

To test these predictions, we assessed the association between reflectivity and microclimate within a phylogenetic framework. First, we quantified UV-visible, NIR and total reflectivity for 19 intertidal gastropod species. Then we conducted three separate phylogenetic comparative analyses, one for each waveband (UV-visible, NIR and total), to determine how microhabitat (exposed versus sheltered) and shell state (wet versus dry) correlate with reflectivity. For the NIR reflectivity model, we accounted for the correlation between NIR and UV-visible reflectivity so we could identify trends in NIR reflectivity independently of UV-visible variation. We also conducted a field experiment to assess whether variation in reflectance influences heat gain in natural conditions. In this experiment, live gastropods were placed in the intertidal zone in the sun and a thermal imaging camera was used to estimate heat gain after sun exposure for a subset of species. Together these studies demonstrate the relationship between reflectance, microhabitat and heating rate of intertidal gastropods.

## Results

**Figure 1:**
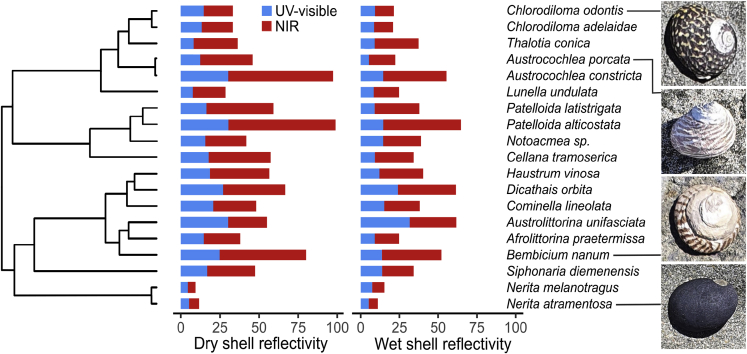
Phylogenetic relationships and reflectivity of intertidal gastropods Bars indicate UV-visible (blue) and near infrared (NIR: red) reflectivity for both dry (left) and wet (right) shells from each species. Images depict *Chlorodiloma odontis, Austrocochlea porcata, Bembicium nanum* and *Nerita atramentosa* (top to bottom). Total reflectivity can be roughly calculated as the average of NIR and UV-visible reflectivity. See Figure S2 for full tree.

We recorded substantial variation in reflectivity across the 19 species measured. Total reflectivity varied from 4.8% for dry *Nerita melanotragus* shells to 49.3% for dry *Patelloida alticostata* shells. NIR reflectivity was more variable than UV-visible reflectivity, ranging from 5–69% compared to 4–30%, respectively (Figure 1). We detected a moderate correlation between NIR reflectivity and UV-visible reflectivity (r^2^ = 0.52, p< 0.001), but there was considerable variation in NIR reflectivity independent of UV-visible reflectivity. The residuals from the linear regression between UV-visible and NIR reflectivity varied from −23.3 for dry *Austrolittorina unifasciata* shells to 21.0 for dry *P. alticostata* shells. This indicates around 40% variation in NIR reflectivity for a given value of UV-visible reflectivity.

**Figure 2:**
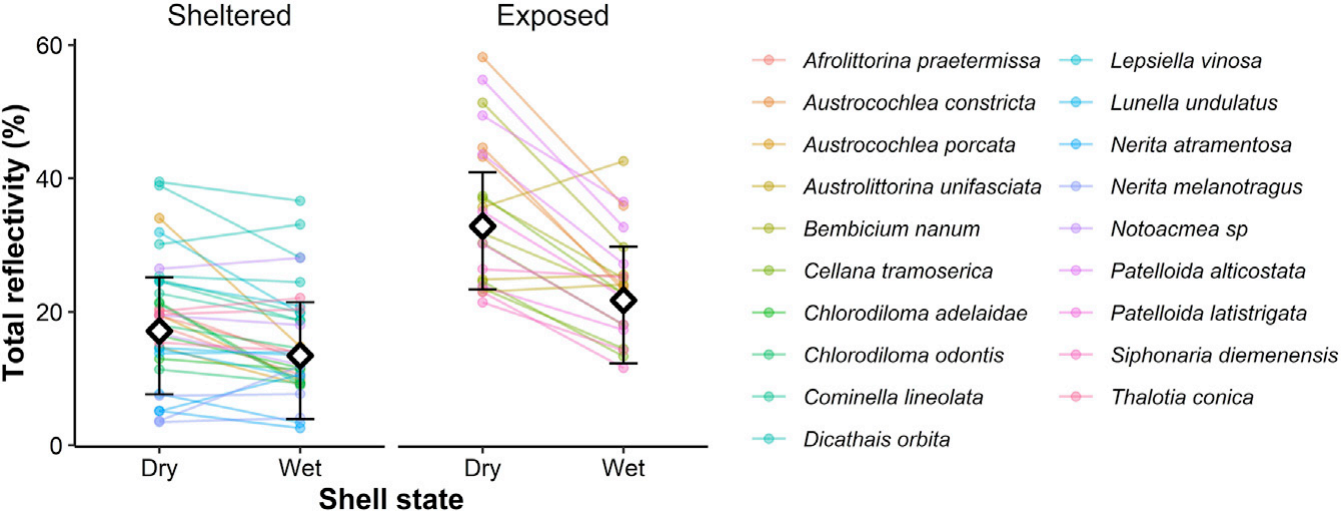
Shell state and position in the intertidal correlate with total reflectivity Exposed gastropods have greater reflectivity than sheltered snails and dry shells have greater reflectivity than wet shells. The difference between dry and wet shells is greater for exposed than sheltered gastropods. Color indicates individuals from the same species and lines join wet and dry measurements from the same individual. Diamonds indicate parameter estimates (mean of posterior distribution) and 95% CI for each group.

We found that total reflectivity was highest for dry shells of species from exposed microhabitats (Figure 2; Table 1). Dry shell total reflectivity for gastropods from exposed habitats was double the total reflectivity for gastropods from sheltered habitats (33.3 versus 16.82%, on average). Although dry shells were lighter than wet shells for both sheltered and exposed gastropods, the increase in total reflectivity from wet to dry was substantially greater for exposed gastropods (Figure 2, Table 1). Results for UV-visible reflectivity were qualitatively the same as those for total reflectivity (Table 1). If reflectance is selected for thermoregulatory functions, we would expect NIR reflectivity to vary with sun exposure even after controlling for UV-visible reflectivity. Our results support this prediction. Specifically, NIR reflectivity of dry shells of exposed gastropods is roughly 4% higher than wet shells of exposed gastropods and 9% higher than wet or dry shells of gastropods from sheltered habitats (Table 1).

**Table 1:** Summary of the results from the phylogenetic mixed models assessing the relationship between microhabitat (sheltered or exposed), shell state (wet or dry) and reflectivity (total, UV-visible or NIR)

	Parameter	Estimate	95% Credibility Interval	ESS
**Total reflectivity**				

Intercept		13.11	3.04, 21.32	2856
Habitat (Exposed)		9.04	1.63, 16.12	3396
State (Dry)		3.71	1.46, 5.83	8483
Habitat (Exposed):State (Dry)		7.44	3.94, 10.97	8679

**UV-Visible reflectivity**				

Intercept		9.56	1.99, 16.00	2754
Habitat (Exposed)		5.26	−0.62, 11.06	3100
State (Dry)		2.71	0.86, 4.58	6746
Habitat (Exposed):State (Dry)		5.42	2.47, 8.41	7004

NIR reflectivity				

Intercept		23.72	10.37, 35.49	3452
UV-Visible reflectivity		1.26	1.08, 1.43	6236
Habitat (Exposed)		6.34	−2.54, 14.80	5764
State (Dry)		1.35	−0.07, 2.77	6448
Habitat (Exposed):State (Dry)		2.70	0.32, 5.12	6104

The intercept refers to wet, sheltered gastropods and parameters show differences between groups. For each parameter included in the model, estimates (mean of the posterior distribution) and 95% credibility intervals are shown. ESS indicates effective sample size.

**Figure 3:**
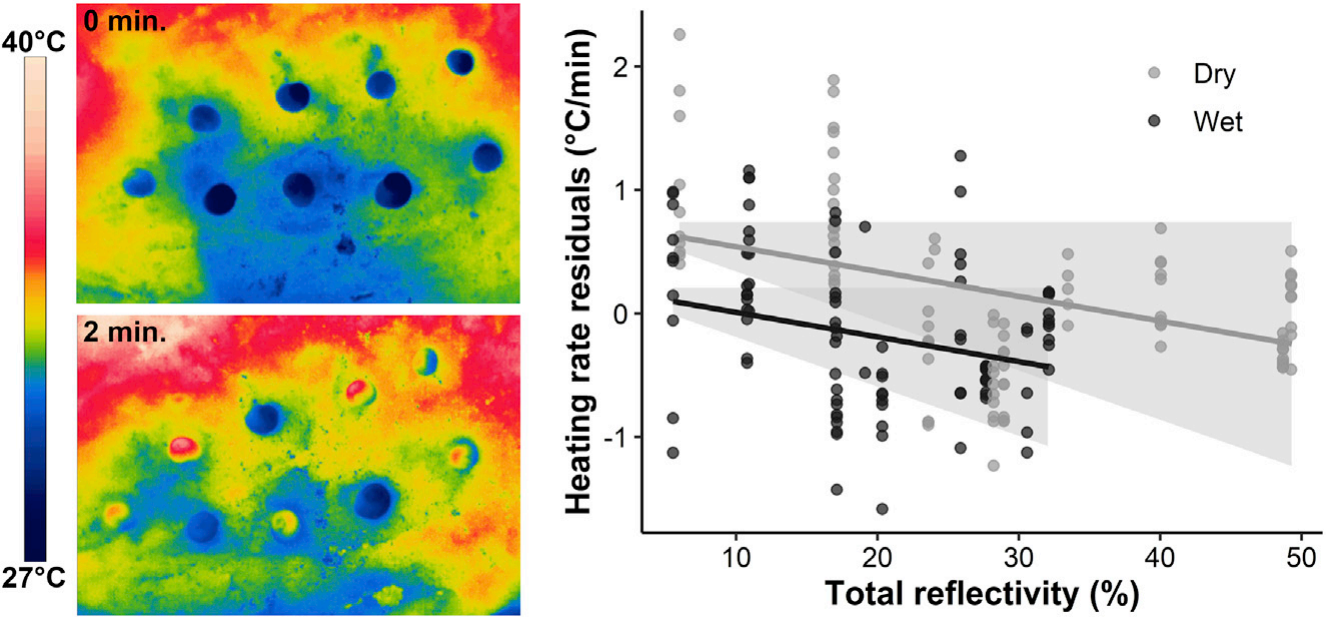
Field experiment investigating the effect of total reflectivity on heating rate for intertidal gastropods with wet or dry shells Left) Thermal images of *C. odontis* taken at 0 and 2 min after exposure to solar radiation. Gastropods are the darker circles in the top photo and are in similar positions in the bottom photograph. See Dryad for all images. Right) Relationship between heating rate and reflectivity. Heating rate residuals are calculated from a linear regression between heating rate and rock temperature to account for conductive heat gain. Trend lines and shaded regions show estimates and 95% credible intervals from the phylogenetic mixed model. Total reflectivity is calculated for each gastropod from 300–1700 nm. Each cluster of dots at the same reflectivity are individual gastropods from the same species (11 species total).

In our heating rate experiment conducted in the intertidal zone, our results suggest that a 10% increase in reflectivity can reduce heating rate by 0.2°Cmin^−1^ (95% CI: −0.4–0.0°Cmin^−1^; Figure 3; Table 2). Across the range of gastropod shell total reflectivity recorded in our field experiment, this trend indicates that low reflectivity gastropods (∼10% total reflectivity) are 1.6°C warmer after 2 min than high reflectivity gastropods (∼50% total reflectivity). As expected, gastropods with wet shells had a lower heating rate than those with dry shells (Table 2). For a given value of nearby rock temperature, the heating rate for gastropods with wet shells was 0.46°Cmin^-1^ lower than that of gastropods with dry shells (Figure 3). There was also an effect of shell length on heating rate, where a 10 mm increase in shell length reduced heat gain by 0.2°Cmin^−1^ (95% CI: −0.3–0.0°Cmin^−1^; Table 2).

**Table 2:** Summary of the results from the phylogenetic mixed models assessing the relationship between total reflectivity, shell length, shell state (wet or dry) and heating rate

Parameter	Estimate	95% Credibility Interval	ESS
Intercept	0.27	−0.27, 0.78	3555
Total reflectivity	−0.02	−0.04, 0.00	3359
Shell length	−0.02	−0.03, 0.00	9442
State (Wet)	−0.46	−0.73, −0.17	3501
Total reflectivity:State (Wet)	0.00	−0.01, 0.02	4672

The intercept refers to dry, sheltered gastropods. For each parameter included in the model, estimates (mean of the posterior distribution) and 95% credibility intervals are shown. ESS indicates effective sample size.

## Discussion

Intertidal gastropods can be exposed to extreme heat during summer low tides.^21^^,^^31^ Adaptations to reduce heat gain are critical for survival, particularly for those species that have low mobility or remain exposed to solar radiation.^5^^,^^6^ Here, we have demonstrated that these gastropods from exposed habitats have higher dry shell reflectivity than those from sheltered habitats, and that the increase in reflectivity as shells dry is larger for gastropods from exposed habitats. We also detected that dry shells of gastropods from exposed habitats have higher NIR reflectivity, even after controlling for UV-visible reflectivity. This suggests that thermoregulation is a key driver of high reflectance for intertidal gastropods in more exposed microhabitats. Our field study provides further support, demonstrating that high total reflectivity can reduce heat gain in natural conditions. Together, these results indicate that reflectivity contributes to thermoregulation for gastropods exposed to solar radiation during summer low tides. More broadly, we show that animal reflectance in both the UV-visible and NIR wavelengths can be driven by the microhabitat of a species.

We found that exposed intertidal gastropods have higher reflectivity in both UV-visible and NIR wavebands. Furthermore, after accounting for UV-visible reflectivity, dry shells of exposed gastropods also had higher NIR reflectivity. These differences were quite substantial, with total reflectivity of gastropods from exposed microhabitats double the total reflectivity of gastropods from sheltered microhabitats, indicating that exposed gastropods are lighter in color than sheltered gastropods. These results are consistent with previous research in terrestrial and intertidal gastropods demonstrating that intraspecific variation in human-visible reflectance can correlate with habitat.^29^^,^^32^^,^^33^^,^^34^ For example, pale morphs of the terrestrial gastropods *Theba pisana* and *Cepaea nemoralis* are more common in open habitats and darker morphs are more common in shady habitats.^32^^,^^33^ More broadly, color morphs may be associated with climate, where darker morphs are more common at higher latitudes (e.g., intertidal snail, *Littorina obtusata*^28^) or in colder climates (e.g., intertidal snail, *Batillaria attramentaria*^30^; terrestrial snail, *T. pisana* (Köhler et al. 2021)^34^). However, these correlations could be driven by thermal requirements, visual selection by predators, or both.^27^^,^^33^^,^^35^^,^^36^ Although both visible and NIR reflectance contribute to thermoregulation, visible reflectance will also be influenced by other functions, such as camouflage or communication. NIR reflectance, however, will be primarily driven by thermoregulatory requirements because most animals cannot see NIR wavelengths.^15^ Therefore, by investigating the relationship between NIR reflectivity and microhabitat, we can determine whether thermoregulation or other functions may be driving patterns of reflectance. By doing this, we found that dry shells of gastropods from exposed habitats have higher NIR reflectivity than would be expected based on UV-visible reflectivity, suggesting that NIR reflectance is selected for thermal requirements. This provides clear support for a thermal function of reflectance in intertidal gastropods.

Exposed gastropods also showed a greater increase in reflectance as the shell dried. This result likely reflects a difference in shell roughness between gastropods in exposed or sheltered microhabitats. Surface roughness influences the effect of water on total reflectance; wet rough surfaces will appear darker than wet smooth surfaces of the same material. This is because the rough surface reflects light diffusely leading to total internal reflection at the liquid-air interface (i.e., the thin film of water on the shell surface).^23^^,^^24^ Anecdotally, we noticed that gastropods in our study with smoother shell surfaces tended to be found in sheltered microhabitats or lower in the intertidal zone (e.g., *Lunella undulatus*, *Thalotia conica*). This observation aligns with previous research indicating that gastropods with rougher or more sculptured shells are often found in more exposed microhabitats.^37^ Increased surface sculpturing or roughness may help keep gastropods cool by increasing surface area for convective heat loss^6^^,^^38^ or trapping more water in the shell to increase evaporative heat loss.^39^^,^^40^ However, on warm to hot days, shells exposed to the sun dry relatively quickly. At this point, gastropods from exposed habitats will benefit from having higher dry shell reflectivity to help reduce heat gain.

Our field experiment indicates that variation in reflectivity impacts heat gain in natural conditions. Gastropods with higher reflectivity shells likely had a lower heating rate than those with lower reflectivity shells (a reduction in heating rate from 0 to 0.4°Cmin^−1^ per 10% increase in reflectivity is reasonably likely). This aligns with previous studies demonstrating that darker morphs can reach higher body temperatures than lighter morphs.^36^^,^^38^^,^^41^ The magnitude of the temperature difference between dark and light morphs varies across studies, from 0.5°C^28^^,^^38^ to up to 3°C,^36^^,^^41^ and can lead to higher mortality for darker morphs in exposed microhabitats.^28^^,^^36^ The magnitude of our results is comparable to these studies, with a difference of roughly 1.6°C between the darkest and lightest species after 2 min. However, our study measured the initial heating rate whereas previous studies have recorded body temperature over several hours. Differences between species may change if we recorded steady state temperature, rather than heat gain.^42^ Nonetheless, our study and these previous studies suggest that reflectance influences body temperature of intertidal gastropods in natural conditions.

Heating rate also varies considerably within each species, likely because of individual behavior or morphology and exposure to different microhabitats. In our study, gastropods showed intraspecific size variation and smaller individuals heated up more quickly than larger individuals. This result aligns with previous research^25^^,^^27^ and is because smaller gastropods have lower thermal inertia. Rock temperature also varied by up to 4°C (range: 0.7–4.2°C) within a photograph and this may influence conduction among individuals. Conduction depends on the area in contact with the rock^25^ and this likely varies among individuals because of behavior (e.g., retraction of the foot) and size. Incorporation of nearby rock temperature into the statistical model may not fully account for this variation. Evaporation may also vary among individuals if individuals use different behavioral responses to increase or decrease evaporative water loss.^29^^,^^37^ Intraspecific variation in reflectivity may also contribute to variation in heating rate. Although our study focused on interspecific variation, and intraspecific variation in reflectivity was substantially smaller than interspecific variation, these differences among individuals may influence radiative heat gain. Further investigation into how these factors interact to influence heat gain in natural conditions is worthwhile.

Reflectivity is only one trait out of numerous morphological, physiological, and behavioral traits that gastropods can use to modulate heat gain.^27^^,^^43^ Shell characteristics (e.g., surface sculpturing, globosity) can influence surface area for absorption of solar radiation or convective heat loss,^6^^,^^37^^,^^44^ shell water retention for evaporative heat loss^39^^,^^40^ and internal volume of fluid to withstand desiccation.^37^ In addition, gastropods exposed to higher microhabitat temperatures often have higher tolerance of these temperatures (i.e. higher lethal temperature^45^^,^^46^). This is likely because of physiological differences, such as in synthesis of heat shock proteins,^45^ lower metabolic rate,^47^ or thermal stability of enzymes.^46^ Behavior also plays a critical role in thermoregulation for gastropods. This includes moving to a cooler microhabitat, (e.g., crevices, under rocks^27^^,^^48^), postures to reduce contact with the substrate (e.g., foot withdrawal, shell lifting, shell standing or shell stacking^48^^,^^49^^,^^50^^,^^51^), changing shell orientation to minimize surface area exposed to the sun,^5^^,^^48^^,^^52^ or forming aggregations to reduce water loss.^53^^,^^54^ These behaviors can have a greater impact on lowering body temperature than morphological adaptations, including reflectivity (e.g., behavioral adaptations: 2–4°C; reflectivity: 0.2–2°C^38^). However, when exposed to extreme temperature during summer low tides, it is likely that behavioral, morphological and physiological adaptations all contribute to maintaining body temperature below lethal limits.

More broadly, our study contributes to growing evidence that reflectivity, and specifically NIR reflectivity, plays an important role in thermoregulation for many animals. Currently, studies including NIR reflectance are limited to insects, including butterflies^12^^,^^13^ and beetles,^8^ and birds.^17^ These studies indicate that higher NIR reflectance can reduce heating rates^8^ and that butterflies or birds in warmer habitats tend to have higher NIR reflectance.^12^^,^^13^^,^^17^ NIR reflectivity data are a valuable addition to these studies to distinguish thermal drivers of reflectance variation from other drivers, such as camouflage or communication.^9^ However, there are also inconsistencies in the relationships reported. For example, higher NIR reflectance is associated with larger Australian butterflies but smaller European butterflies. This difference is likely to reduce overheating in larger Australian butterflies and increase warming in larger European butterflies.^12^^,^^13^ Such results demonstrate the need to investigate drivers of NIR reflectance in more taxa and across different habitats. Here, we expand on these studies to show that within a habitat, NIR and total reflectance can vary depending on the microhabitat of the species. Together, these studies provide growing evidence that NIR reflectance is a critical component of thermoregulation for many species exposed to high levels of solar radiation.

### Limitations of the study

Our study demonstrated that interspecific variation in reflectance is associated with microhabitat and that this variation influences heat gain. We did not assess intraspecific variation in reflectance among gastropods and this may be a fruitful avenue for future studies. In addition, to detect variation in radiative heat gain among gastropods our field study was conducted on a warm day with minimal wind. Under other conditions, such as high winds or cloudy days, radiative heat gain may have a small effect compared to convection, conduction or evaporation.

## STAR★Methods

### Key resources table

**Table:** 

REAGENT or RESOURCE	SOURCE	IDENTIFIER
**Biological samples**		

*Afrolittorina praetermissa*	This study	
*Austrocochlea constricta*	This study	
*Austrocochlea porcata*	This study	
*Austrolittorina unifasciata*	This study	
*Austromytilus rostratus*	This study	
*Bembicium nanum*	This study	
*Cellana tramoserica*	This study	
*Chlorodiloma adelaidae*	This study	
*Chlorodiloma odontis*	This study	
*Cominella lineolata*	This study	
*Dicathais orbita*	This study	
*Haustrum vinosa*	This study	
*Lunella undulata*	This study	
*Nerita atramentosa*	This study	
*Nerita melanotragus*	This study	
*Notoacmea sp*	This study	
*Patelloida alticostata*	This study	
*Patelloida latistrigata*	This study	
*Siphonaria diemenensis*	This study	
*Thalotia conica*	This study	

**Deposited data**		

Raw data: spectral reflectance data	This study	Dryad: https://doi.org/10.5061/dryad.905qfttnv
Raw data: Thermal images	This study	Dryad: https://doi.org/10.5061/dryad.905qfttnv

**Software and algorithms**		

R Script to run analyses	This study	Dryad: https://doi.org/10.5061/dryad.905qfttnv
Source code to construct phylogenetic tree	This study	Dryad: https://doi.org/10.5061/dryad.905qfttnv
R v4.0.5	Venables and Smith, 2003	https://www.r-project.org/
FLIR Tools	Teledyne FLIR	https://www.flir.com/support/products/flir-tools/
BEAST	Center for Computational Evolution	https://www.beast2.org/

### Resource availability

#### Lead contact

Further information and requests for resources should be directed to and will be fulfilled by the lead contact, Amanda Franklin (amandaf@unimelb.edu).

#### Materials availability

This study did not generate new unique reagents or material.

### Experimental model and subject details

In this study, gastropods were collected from intertidal shores in southern Australia (Victorian Fisheries Permit: RP1425). Full list of species is in the key resources table and full details of experimental protocols are described below.

### Method details

#### Study sites and species

This study was conducted in January to March 2021 and January 2022 at two rocky intertidal sites in Victoria, Australia: Jan Juc (38°20′45.6”S 144°19’07.4”E) and Cape Patterson (38°40’33.6”S 145°36’56.2”E). Both sites have diverse intertidal communities, including many gastropod species, that can be exposed to high temperatures (>40°C) during summer low tides. At these sites, we collected gastropods to bring back to the laboratory for reflectance measurements and to conduct an *in-situ* heating rate experiment (Victorian Fisheries Permit: RP1425). The microhabitat of all species collected was independently classified by two researchers as either exposed (i.e. on top of rocks, exposed to sunlight) or sheltered (i.e. in crevices, under rock overhangs) based on the majority of individuals on warm to hot (25–30°C) sunny days. These classifications were compared with the literature where possible (Table S1).

#### Reflectivity

We measured UV, visible and NIR reflectance (300–1700 nm; 98.9% of solar energy) of three snails from 19 species (Table S1). Reflectance was measured using a Flame UV/VIS spectrometer (300–1000 nm; Ocean Optics Inc.) and an NIRQuest spectrometer (1000–1700 nm; Ocean Optics Inc.). To provide illumination across the entire spectrum we used both a PX2 pulsed xenon light source (UV and visible light; Ocean Optics Inc.) and a HL-2000 tungsten halogen light source (visible and NIR light; Ocean Optics Inc.). The light source and collector probes were set at 10° and −10° to the normal of the sample, and all measurements were relative to a Spectralon 99% white reflectance standard (LabSphere, NH, USA). Whilst an integrating sphere would provide a more accurate measurement of reflection across all angles,^55^ most of the shells were too small and curved for the sampling area of an integrating sphere (commonly 4mm or greater). Our set-up allows measurement of a small area (∼1 mm) but assumes that shell surfaces are diffuse. To confirm this, for a subset of four species we measured reflectance of wet and dry shells using a specular and non-specular geometry of the light source and collector probe. We found that measurement geometry did not influence reflectivity (F = 0.0001, df = 1, p = 0.99) and that geometry did not differentially affect reflectivity depending on shell state (F = 0.18, df = 1, p = 0.68), suggesting that both wet and dry shells are diffuse (Figure S1).

For each snail, we recorded reflectance of the shell when wet and dry. To measure the reflectance of a wet shell, seawater was dripped on the shell, left to soak into the shell for several seconds and then excess water blotted off. For species with patterns larger than 1 mm (i.e. the size of the sample area), we measured the reflectance of each colour patch. All spectral data were smoothed in R using the *pavo* package^56^ to remove noise (procspec function, span = 0.10).

Heating rate is proportional to the total amount of energy absorbed by a surface (i.e. the energy that is not reflected or transmitted) and depends on the reflectance spectrum of the surface as well as the irradiance spectrum of the light source.^8^^,^^14^ Therefore, we used the reflectance measurements and solar spectral irradiance (ASTM G-173^57^^,^^58^) to calculate reflectivity of each colour patch. Reflectivity is the ratio of incident light to reflected light, integrated over the wavelength range of solar radiation (300–2600 nm).^55^ Specifically, reflectivity (R) is calculated using Equation 1,^14^

(Equation 1) $$R=\frac{\int_{i}^{n}S\left(\lambda \right)I\left(\lambda \right)d\lambda }{\int_{i}^{n}I\left(\lambda \right)d\lambda }$$

where S is the reflectance of the shell and I is the solar irradiance across wavelengths λ from i to n. We calculated reflectivity from 300–1700 nm, which accounts for roughly 98.9% of incident solar radiation.^8^ For each colour patch on each snail, we calculated reflectivity for the total range (300–1700 nm), UV-visible wavelengths (300–700 nm) and NIR wavelengths (700–1700 nm). Within the wavelength range we assessed, roughly 50.3% is UV-visible light and 49.7% is NIR light. Therefore, total reflectivity could be roughly calculated as (UV-visible + NIR)/2. For snails with more than one colour patch, each colour covered an approximately equal area of the shell. Therefore, we averaged the reflectivity measurements from each colour patch to obtain one measure of shell reflectivity. For all individuals measured we had one wet and one dry reflectivity measurement for each wavelength range.

#### Heating rate experiment

To determine whether reflectivity may influence heat gain in natural conditions, we conducted a field experiment at Cape Patterson (38’40′3”.6"S 145’36′5”.2"E) during low tide (1030–1330) on a warm sunny day (22–26°C). We collected individuals from 11 species that represented a range of low to high reflectivity (Table S1). Where possible, we collected 10 individuals, but for *Cominella lineolata*, *Dicathais orbita*, *Chlorodiloma adelaidae* and *Chlorodiloma odontis* we could only find two, five, nine and nine individuals, respectively. Upon collection, shells were dried with paper towel and snails were placed in a white bucket in the shade for a minimum of five minutes to stabilise temperature. To begin the experiment, snails were placed on a flat section of rock and half had water dripped over their shells whilst the other half remained dry. A thermal image was recorded using a FLIR thermal camera (T420, Teledyne FLIR, USA) at the beginning of exposure to the sun and after 2.5 min of sun exposure (range: 135–195s). Following this, gastropods were dried and placed in the shade again before repeating the experiment with treatment reversed for each individual.

Images were processed using FLIR Tools software. From the images, we calculated the average surface temperature of the shell (emissivity: 0.95^27^) because the average shell surface temperature has been shown to correlate more closely with body temperature of gastropods than maximum surface temperature.^27^ To calculate heating rate for each gastropod, we calculated the difference between average shell temperature at the beginning and end of solar radiation exposure divided by the time between photos. We also calculated the average rock temperature for each snail by selecting a patch of rock near each snail and calculating the average temperature using both the beginning and end photographs (emissivity of rough sandstone: 0.935^59^).

#### Phylogenetic supermatrix assembly

We compiled a species-level supermatrix of geneticdata using i) a core framework derived from the transcriptome phylogenomic study of Cunha and Giribet,^60^ and ii) sequence data downloaded from NCBI (https://www.ncbi.nlm.nih.gov/) based on the ‘Organism’ field for six genes with suitable taxon coverage and phylogenetic signal. Table S2 provides detailed information on accessions and data. Taxonomy and nomenclature were based on MolluscaBase (https://www.molluscabase.org/).

The large Gastropod amino acid phylogenomic dataset was compacted down by jack-knifing the 1059 loci, favouring more complete sites, from 362,493 amino acid sites (0.46 complete) to 5,810 (0.79 complete).

Geneticdata was aligned with MAFFT (v. 7.245)^61^ using the local-pair (L-INS-i) algorithm, alignments assembled into a custom Microsoft Excel database, and nomenclature rationalized. Gene trees were inferred by IQ-TREE^62^ using models of sequence evolution identified by ModelFinder as implemented in IQTREE,^63^ and aberrant accessions excluded from the database. Some Trochidae 18S was removed to ensure trochid monophyly. Concatenated supermatrix sequence data then used the best (longest accepted) single exemplar sequence per gene per taxon, and gene partitions compacted by removing regions with little or no data and ambiguous alignment (<33% taxa with any data per gene; custom scripts see Dryad: https://doi.org/10.5061/dryad.905qfttnv, PAT_script_v5; Aliscore v2.2).^64^

We formed a maximally filled composite datamatrix with 7 ‘genes’ (including a phylogenomic block of nuclear exon amino acid sequence), substituting data to create chimeric higher rank representatives where suitable (i.e. the only taxon in that rank with that gene data in our final tree). In total 29 accessions (out of 94) were substituted, 22 by genus and 7 by family. This gave a final matrix of 19 species of 11,123 characters (sites) mean 49% complete (and 65% complete for the DNA section; see Table S2 for details).

#### Phylogenetic inference

Bayesian relaxed-clock analyses were performed in BEAST v2.4^65^ with i) lognormal clock or ii) random local clock, and Yule speciation prior (with 1/X parameterization), using a partition sequence evolution model optimized by ModelFinder. Full details on the data, model and priors are available in the xml file on Dryad: https://doi.org/10.5061/dryad.905qfttnv, GASTRO_mat1pd_bst2xc.xml. The analyses were rooted (and root age prior set) according to the results of Cunha and Giribet^60^ and Zapata et al.^66^ We ran two independent Bayesian MCMC analyses of 50 million steps sampling every 5,000, with a burn-in of 20% that returned all parameters with ESS >500.^67^ Runs were then pooled to compute a maximum clade credibility (MCC) tree (Figure S2). For the purposes of comparative analyses we used the random local clock tree.

### Quantification and statistical analysis

We ran phylogenetic generalized mixed models to investigate whether reflectivity correlated with shell state (wet or dry) and gastropod microhabitat (exposed or sheltered). Three models were run, one for each wavelength range (total, UV-visible or NIR). All models included shell state, microhabitat, and the interaction between these two factors as fixed effects. We also included individual ID and species ID as random effects to account for multiple measurements within individuals (wet vs dry) and species. UV-visible and NIR reflectance are part of the same continuous spectrum and can be highly correlated. In our dataset, UV-visible and NIR reflectance were moderately correlated (r^2^ = 0.52, p< 0.001). This correlation can influence the results for NIR reflectivity and needs to be accounted for in the statistical model. Previous studies have either calculated residuals from a linear model between NIR reflectivity and UV-visible reflectivity, or included UV-visible reflectivity as a covariate in the model.^12^^,^^13^ Here, we included UV-visible reflectivity as a covariate in the statistical model with NIR reflectivity as the response variable. However, we also ran the analysis with the residuals from a regression of NIR reflectivity against UV-visible reflectivity as the response variable and results were qualitatively similar (Table S3). For further comparison of the differences between these model structures, see discussion provided by Kang and colleagues.^13^ For the UV-visible and total analyses, UV-visible reflectivity and total reflectivity were the response variables, respectively.

We also ran a phylogenetic generalized mixed model to investigate whether the shell reflectivity influences heating rate in natural conditions. During this experiment, heating rate of each snail will be affected by conduction from the rock and radiation from the sun. To determine whether reflectivity influences heat gain, we accounted for conductive heat gain by calculating the residuals from a linear regression between heating rate and rock temperature. Therefore, for this model we included heating rate residuals as the response variable and total reflectivity, shell state (wet or dry), shell length (mm) and the interaction between reflectivity and shell state as predictor variables. Shell length was included as a predictor variable because body size can influence heating rate.^27^ We also included species ID and individual ID as random effects to account for multiple measurements within a species and individuals.

The models were run in R v4.1.2^68^ using the brms package,^69^which uses Markov chain Monte Carlo (MCMC) sampling to obtain draws from posterior distributions. All models were run using the brm() function with a Gaussian family and weak priors (intercept: normal(mean = 0, sd = 10); slope: normal(mean = 0, sd = 50); standard deviation: student_t(df = 3, mean = 0, sd = 20); sigma: student_t(df = 3, mean = 0, sd = 20)). We ran the analyses for two chains with 6000 iterations and discarded the first 2000 as burn-in. This ensured the final effective sample size for each parameter was above 2000 to avoid autocorrelation. To assess convergence, we visually examined trace plots and confirmed all $\hat{R}$ values equalled 1. All models were run twice more with different priors (see R script on Dryad: https://doi.org/10.5061/dryad.905qfttnv) to confirm that selection of priors did not influence the results. We interpret results by assessing the effect sizes and 95% credible intervals to provide more information than only stating p-values.^70^^,^^71^^,^^72^ Effect sizes and 95% credible intervals provide an indication of the magnitude of the effect as well as the uncertainty in our estimate, rather than only whether a factor is significant or not. To assess results in this way, the parameter estimate provides an estimate of the effect size and the credible interval provide the range of effect sizes that are reasonably likely.

## Data Availability

• Data are available from Dryad: https://doi.org/10.5061/dryad.905qfttnv and will be publicly available as of the date of publication. DOIs will be listed in the key resources table. • Analysis scripts are available from Dryad: https://doi.org/10.5061/dryad.905qfttnv and will be publicly available as of the date of publication. DOIs will be listed in the key resources table. • Any additional information required to reanalyse the data reported in this paper is available from the lead contact upon request.
