# Supplementary material for: Exposure to thermal extremes favors higher solar reflectivity in intertidal gastropods
Source: iScience. 2022 Nov 25;25(12):105674. doi: 10.1016/j.isci.2022.105674 (PMC9758529; doi:10.1016/j.isci.2022.105674)
Supplement: Document S1. Figures S1 and S2 and Tables S1–S3 [file mmc1.pdf]

iScience, Volume *25*

Supplemental information

Exposure to thermal extremes favors higher solar reflectivity in intertidal gastropods

Amanda M. Franklin, Katrina J. Rankin, Andrew Hugall, and Devi Stuart-Fox

Supplementary Information

Table S1: Details of species collected. All species were used to investigate whether reflectivity correlated with microhabitat and a subset were used to investigate heat gain in the field (indicated in "Field experiment" column). Refs provides details of field guides used to assess biology and behaviour of each species, in addition to our field observations. Related to Figure 2.

Family	Species	Intertidal location	Microhabitat	Field experiment	Refs.
Littorinidae	*Afrolittorina praetermissa*	High	sheltered		[1, 2]
Trochidae	*Austrocochlea constricta*	High	exposed	Y	[1-3]
Trochidae	*Austrocochlea porcata*	High	sheltered		[1]
Littorinidae	*Austrolittorina unifasciata*	High	exposed		[1, 2, 4]
Muricidae	*Bedeva vinosa*	Low	sheltered	Y	[1, 2]
Littorinidae	*Bembicium nanum*	High	exposed	Y	[1, 2, 5]
Nacellidae	*Cellana tramoserica*	High	exposed	Y	[1, 2, 6]
Trochidae	*Chlorodiloma adelaidae*	Low	sheltered	Y	[1]
Trochidae	*Chlorodiloma odontis*	Low	sheltered	Y	[2]
Buccinidae	*Cominella lineolata*	Low	sheltered	Y	[1, 2, 7]
Muricidae	*Dicathais orbita*	High	sheltered	Y	[1, 2, 8]
Turbinidae	*Lunella undulatus*	Low	sheltered		[1, 2, 9]
Neritidae	*Nerita atramentosa*	High	sheltered	Y	[1, 2, 10]
Neritidae	*Nerita melanotragus*	High	sheltered		[11]
Lottiidae	*Notoacmea sp*	High	sheltered		[2]
Lottiidae	*Patelloida alticostata*	High	exposed	Y	[1, 2]
Lottiidae	*Patelloida latistrigata*	High	exposed		[2]
Siphonariidae	*Siphonaria diemenensis*	High	exposed	Y	[1, 2, 12]
Trochidae	*Thalotia conica*	Low	sheltered		[1]

1. Gowlett-Holmes, K. (2008). *A field guide to the marine invertebrates of South Australia*. Sandy Bay, Tas: Notomares.
2. Museums Victoria (2014). *Museums Field Guide Apps, Species Profiles – marine invertebrates*. Accessed: 24 May 2022; Available from: https://lists.ala.org.au/speciesListItem/list/dr1147.
3. Patullo, B. (2011). *Ribbed top shell, Austrocochlea constricta*. Taxonomic Toolkit for marine life of Port Phillip Bay. Accessed: 24 May 2022; Available from: https://portphillipmarinelife.net.au/species/10933.
4. Patullo, B. (2011). *Marine snail, Austrolittorina unifasciata*. Taxonomic Toolkit for marine life of Port Phillip Bay. Accessed: 24 May 2022; Available from: https://portphillipmarinelife.net.au/species/10941.
5. Patullo, B. (2011). *Striped-mouth conniwink, Bembicium nanum*. Taxonomic Toolkit for marine life of Port Phillip Bay. Accessed: 24 May 2022; Available from: https://portphillipmarinelife.net.au/species/10934.
6. Patullo, B. (2011). *Common limpet, Cellana tramoserica*. Taxonomic Toolkit for marine life of Port Phillip Bay. Accessed: 24 May 2022; Available from: https://portphillipmarinelife.net.au/species/10931.
7. Patullo, B. (2011). *Lineated cominella, Cominella lineolata*. Taxonomic Toolkit for marine life of Port Phillip Bay. Accessed: 24 May 2022; Available from: https://portphillipmarinelife.net.au/species/10931.
8. Patullo, B. (2011). *Dog winkle, Dicathais orbita*. Taxonomic Toolkit for marine life of Port Phillip Bay. Accessed: 24 May 2022; Available from: https://portphillipmarinelife.net.au/species/10937.
9. Patullo, B. (2011). *Turban shell, Turbo undulatus*. Taxonomic Toolkit for marine life of Port Phillip Bay. Accessed: 24 May 2022; Available from: https://portphillipmarinelife.net.au/species/10946.
10. Patullo, B. (2011). *Black nerite, Nerita atramentosa*. Taxonomic Toolkit for marine life of Port Phillip Bay. Accessed: 24 May 2022; Available from: https://portphillipmarinelife.net.au/species/10939.
11. New Zealand Marine Studies Centre (2020). *Black nerita snail, Nerita melanotragus*. Marine Life Database. Accessed: 24 May 2022; Available from: https://www.marinelife.ac.nz/species/5296.
12. Patullo, B. (2011). *False limpet, Siphonaria diemenensis*. Taxonomic Toolkit for marine life of Port Phillip Bay. Accessed: 24 May 2022; Available from: https://portphillipmarinelife.net.au/species/10932.

Table S2. Sequence data used to represent the terminal taxa. Related to Figure 1.

Subclass	Family	Genus	Species	exon-AA	H3	18S	28S	COI	12s	16S
Caenogastropoda	Buccinidae	*Cominella*	*lineolata*	--	--	KP694175.1	--	KP694130.1	--	KP694078.1
Caenogastropoda	Littorinidae	*Afrolittorina*	*praetermissa*	--	--	AJ488708.1	AJ488667.1	AJ622950.1	AJ488749.1	--
Caenogastropoda	Littorinidae	*Austrolittorina*	*unifasciata*	**SRR1269556**	**AF033705.1**	**AJ488705.1**	KC109993.1	AY296829.1	DQ916420.1	KC109941.1
Caenogastropoda	Littorinidae	*Bembicium*	*nanum*	--	--	**AJ488688.1**	**AJ488647.1**	FJ516177.1	**AJ488729.1**	--
Caenogastropoda	Muricidae	*Dicathais*	*orbita*	**SRR1505141**	AF033690.1	HM486916.1	EU391553.2	AY296838.1	FN677395.1	--
Caenogastropoda	Muricidae	*Haustrum*	*vinosa*	--	--	**HM486917.1**	--	**FJ516017.1**	**FN677389.1**	**KP845071.1**
Heterobranchia	Siphonariidae	*Siphonaria*	*diemenensis*	**SRR1505107**	**KM040909.1**	**AY427523.1**	**AY465080.1**	**MN690211.1**	**KF001107.1**	**HQ650566.1**
Neritimorpha	Neritidae	*Nerita*	*atramentosa*	--	AF033701.2	AM048633.1	AM048693.1	DQ060937.1	DQ916444.1	EU732060.1
Neritimorpha	Neritidae	*Nerita*	*melanotragus*	SRR1920139	--	**DQ093429.1**	--	EF631986.1	--	EU732112.1
Patellogastropoda	Lottiidae	*Notoacmea*	*petterdi*	--	AF033703.1	--	AH005992.2	AB238502.1	AB238261.1	AB238372.1
Patellogastropoda	Lottiidae	*Patelloida*	*alticostata*	**SRR8318348**	**DQ093492.1**	**AB282757.1**	**AB282786.1**	AB238512.1	AB238271.1	AB238383.1
Patellogastropoda	Lottiidae	*Patelloida*	*latistrigata*	--	--	--	--	--	AF058220.1	AF058269.1
Patellogastropoda	Nacellidae	*Cellana*	*tramoserica*	**SRR8318358**	AB433687.1	**DQ013353.1**	**AB282789.1**	FJ515966.1	AF058218.1	AB238431.1
Vetigastropoda	Trochidae	*Austrocochlea*	*constricta*	--	--	--	GQ249704.1	AY858086.1	GQ249756.1	AY855322.1
Vetigastropoda	Trochidae	*Austrocochlea*	*porcata*	**SRR1505119**	AF033676.1	--	GQ249706.1	AY858087.1	GQ249754.1	AY855323.1
Vetigastropoda	Trochidae	*Chlorodiloma*	*adelaidae*	--	--	--	GQ249712.1	MH221581.1	GQ249759.1	AY855317.1
Vetigastropoda	Trochidae	*Chlorodiloma*	*odontis*	--	--	--	GQ249714.1	AY858082.1	GQ249788.1	AY855318.1
Vetigastropoda	Trochidae	*Thalotia*	*conica*	--	--	--	EU530022.1	FN435322.1	GQ232345.1	GQ232309.1
Vetigastropoda	Turbinidae	*Lunella*	*undulata*	--	**FJ977742.1**	**AY698071.1**	AM403966.1	FR693851.1	FR695572.1	FR694430.1

Taxonomy according to MolluscaBase. NCBI accessions, rank substituted representatives in bold:
exon-AA (5810 sites) Cunha and Giribet (2019) exon amino acid data (SRA accessions but data from the Cunha & Giribet [1] Alignments.tar.gz file at https://doi.org/10.7910/DVN/O85KLQ); **H3** (327 sites) Histone H3; **18S** (1764 sites) ribosomal **18S**; **28S** (1453 sites) ribosomal 28S; **COI** (657 sites) mitochondrial COI; **12S** (563 sites) mitochondrial ribosomal 12S; **16S**: (549 sites) mitochondrial ribosomal 16S

Key references for sequence data:

1. Cunha, T. J., and G. Giribet. 2019. A congruent topology for deep gastropod relationships. *Proceedings of the Royal Society B* 286:20182776.
2. Williams & Ozawa 2006 Molecular phylogeny suggests polyphyly of both the turban shells (family Turbinidae) and the superfamily Trochoidea (Mollusca : Vetigastropoda). *Molecular Phylogenetics and Evolution* 39: 33-51

Table S3: Summary of the results from the phylogenetic mixed model assessing the relationship between microhabitat (sheltered or exposed), shell state (wet or dry) and NIR reflectivity residuals. The response variable for this model was the residuals from a regression of NIR reflectivity against UV-visible reflectivity. The intercept refers to wet, sheltered gastropods and parameters show differences between groups. For each parameter included in the model, estimates (mean of the posterior distribution) and 95% credibility intervals are shown. ESS indicates effective sample size. Related to Table 1.

**Parameter**	**Estimate**	**95% Credibility Interval**	**ESS**
Intercept	-3.50	-16.49, 9.07	6173
Habitat (Exposed)	5.75	-3.29, 14.43	7912
State (Dry)	1.04	-0.29, 2.39	12405
Habitat (Exposed):State (Dry)	2.06	-0.12, 4.26	11076

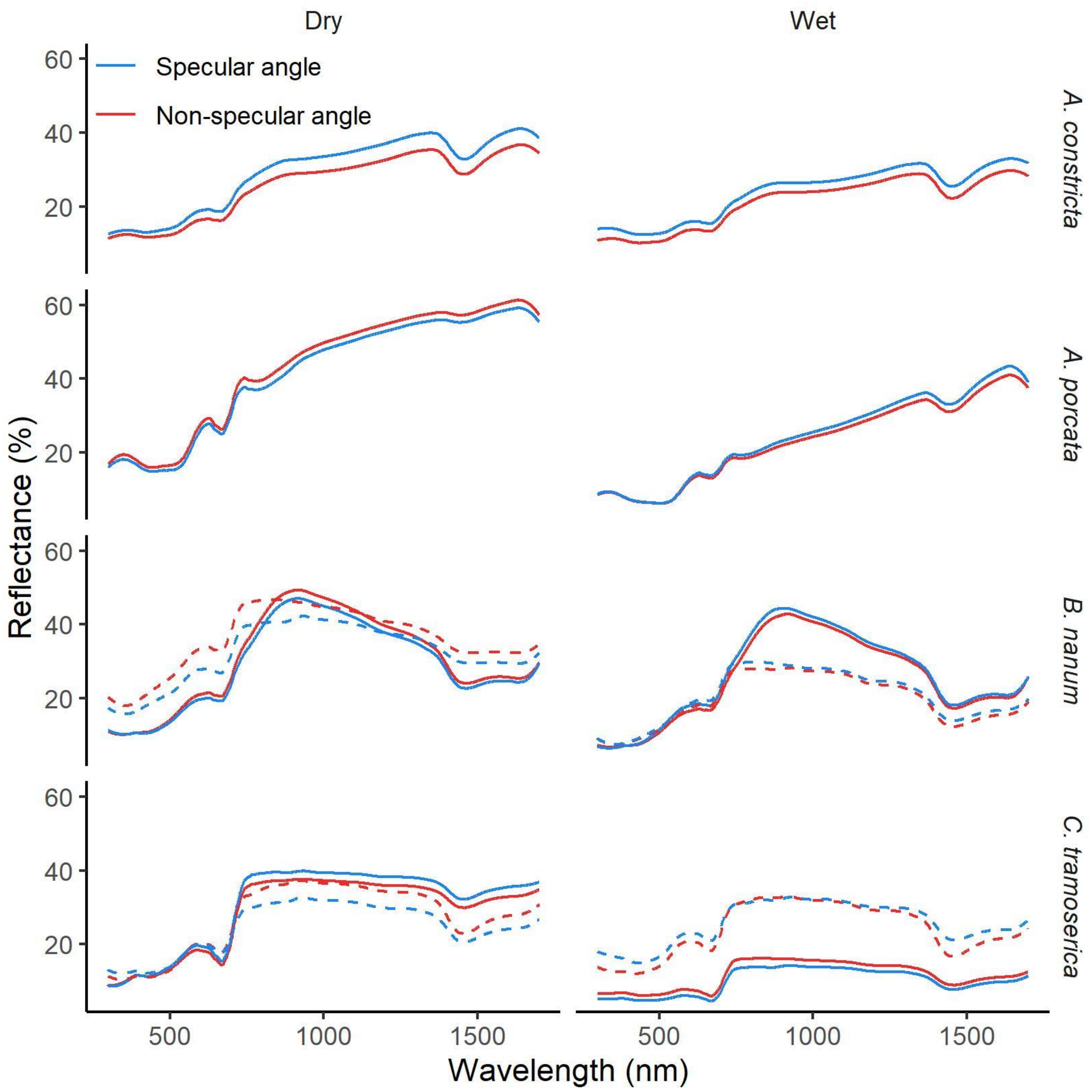

Figure S1: Reflectance of dry and wet shells from four species recorded at the specular angle and away from the specular angle. Specular measurements (blue) were recorded with the light source and collector probes set at 10° and -10° to the normal of the sample and non-specular measurements (red) were recorded with light source and collector probe set at 20° and -10° to the normal, respectively. For *B. nanum* and *C. tramoserica*, two colour patches were measured, indicated by dotted or solid lines. The measurement geometry does not influence reflectance indicating shells are diffuse. Related to Figure 1.

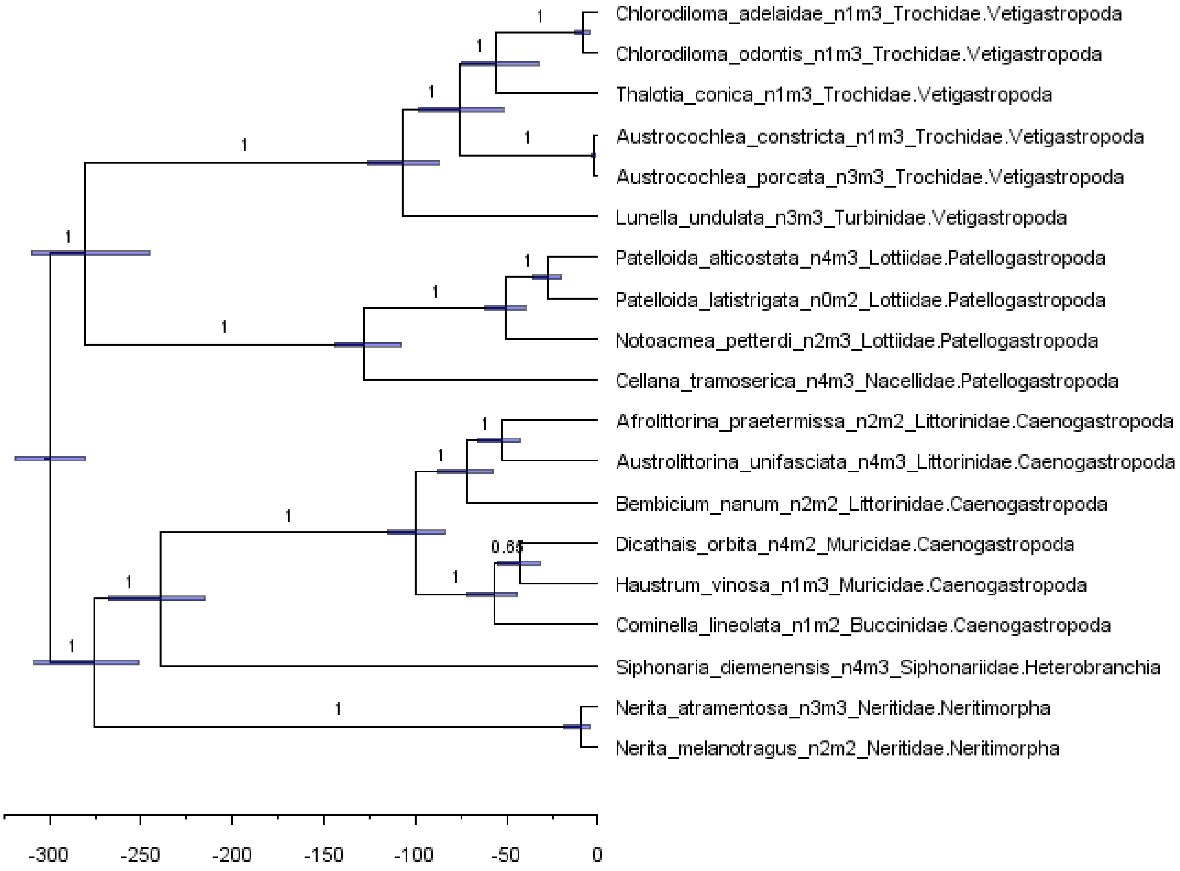

Figure S2: BEAST local random clock Yule model posterior maximum clade credibility tree with node posterior probability support and divergence height 95% confidence intervals. Taxon labels show genus_species_number of nuclear and mitochondrial accessions_family_subclass. See Table S2 for description of genetic data. Related to Figure 1.
